# Aggregation and clogging phenomena of rigid microparticles in microfluidics: Comparison of a discrete element method (DEM) and CFD–DEM coupling method

**DOI:** 10.1007/s10404-018-2124-7

**Published:** 2018-08-30

**Authors:** Khurram Shahzad, Wouter Van Aeken, Milad Mottaghi, Vahid Kazemi Kamyab, Simon Kuhn

**Affiliations:** Department of Chemical Engineering, KU Leuven, Celestijnenlaan 200F, 3001 Leuven, Belgium

**Keywords:** Microfluidics, Clogging, Aggregation, CFD–DEM simulation

## Abstract

We developed a numerical tool to investigate the phenomena of aggregation and clogging of rigid microparticles suspended in a Newtonian fluid transported through a straight microchannel. In a first step, we implement a time-dependent one-way coupling Discrete Element Method (DEM) technique to simulate the movement and effect of adhesion on rigid microparticles in two- and three-dimensional computational domains. The Johnson–Kendall–Roberts (JKR) theory of adhesion is applied to investigate the contact mechanics of particle–particle and particle–wall interactions. Using the one-way coupled solver, the agglomeration, aggregation and deposition behavior of the microparticles is studied by varying the Reynolds number and the particle adhesion. In a second step, we apply a two-way coupling CFD–DEM approach, which solves the equation of motion for each particle, and transfers the force field corresponding to particle–fluid interactions to the CFD toolbox OpenFOAM. Results for the one-way (DEM) and two-way (CFD–DEM) coupling techniques are compared in terms of aggregate size, aggregate percentages, spatial and temporal evaluation of aggregates in 2D and 3D. We conclude that two-way coupling is the more realistic approach, which can accurately capture the particle–fluid dynamics in microfluidic applications.

**Electronic supplementary material:**

The online version of this article (10.1007/s10404-018-2124-7) contains supplementary material, which is available to authorized users.

## Introduction

**Fig. 1:**
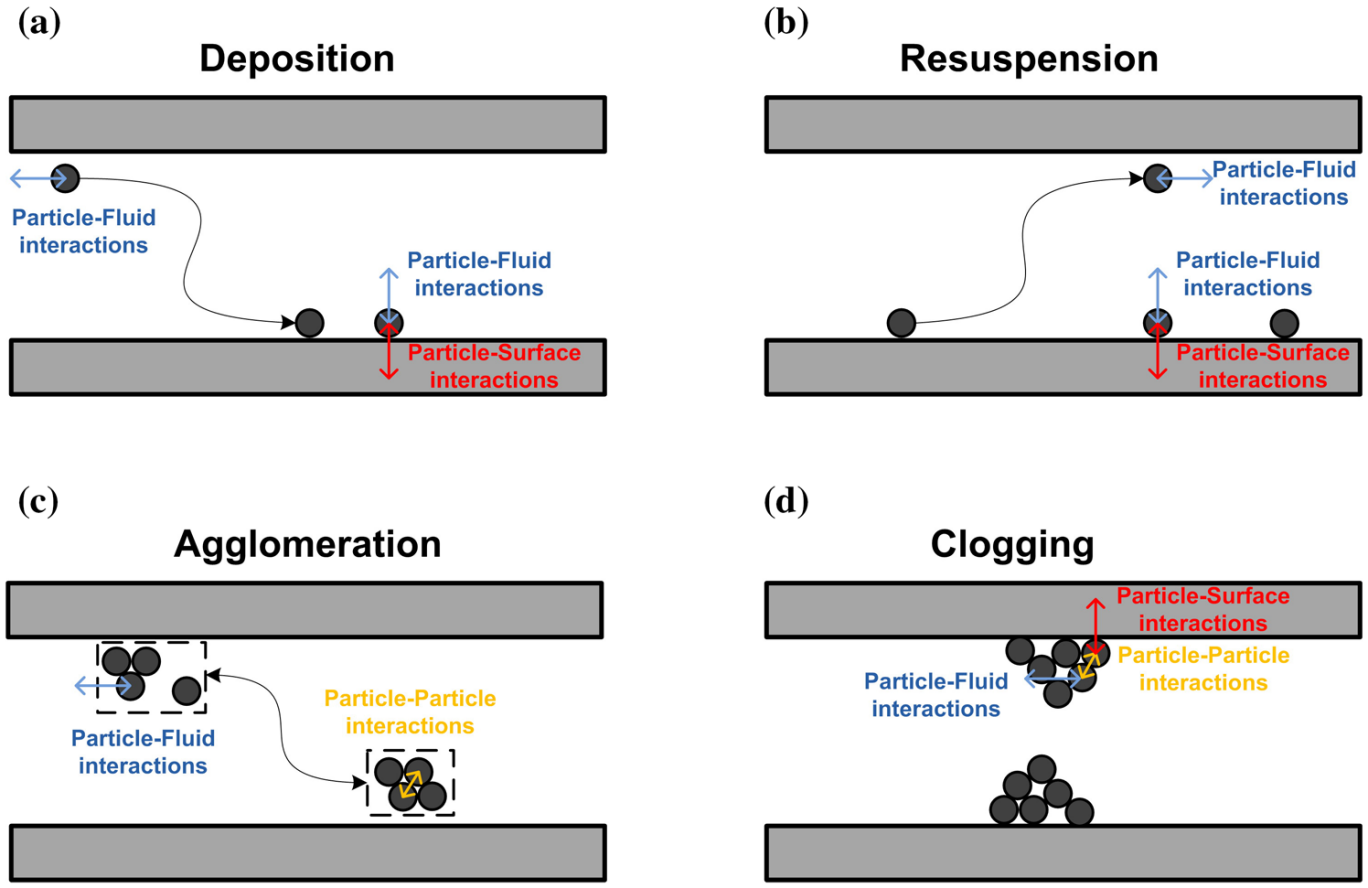
Interactions governing the behavior of solid particles in microchannels (Wu and Kuhn 2014). **a** Deposition of particles is initiated by particle–fluid interactions transporting the solid to the microchannel wall where it finally sticks due to a dominating particle–surface interaction. **b** Increasing the particle–fluid interaction by, e.g., increasing the fluid velocity will lead to resuspension. **c** The particles will agglomerate in the bulk of the fluid by particle–particle interactions; however, agglomerate break-up can again occur when the particle–fluid interactions overcome the inter-particle interactions. **d** The clogging phenomena itself is governed by all three interactions, and usually occurs via bridging of a constricted microchannel cross-section. Reproduced with permission from Wu and Kuhn (2014). Copyright 2014 Tekno Scienze Srl

Microreaction technology has gained popularity and attention beyond the scope of academic labs due to the inherent benefits associated with the decrease in characteristic length scale, such as increased heat and mass transfer, safer handling of hazardous materials, precise control of residence time and reaction conditions (Tabeling 2006). Over the past two decades, this has led to a wide range of commercial equipment and applications (Jensen 2017). However, the use of this technology is still limited by their ability to handle solid material in small-scale devices, which elevates the risk of channel clogging and thus limited life span of these devices. The solid materials can either arise as insoluble starting reagents or products of a reaction, or solid particles can be deliberately added as heterogeneous catalyst for a chemical transformation (Ufer et al. 2011; Liedtke et al. 2015; Schoenitz et al. 2015; Pu and Su 2018). Therefore, the research effort in recent years was dedicated to the experimental investigation of clogging phenomena under various flow conditions in microfluidic devices, and clogging mitigation using external actuation such as ultrasound (Hartman et al. 2010; Kuhn et al. 2011; Hartman 2012; Flowers and Hartman 2012; Wu and Kuhn 2014; Fernandez Rivas and Kuhn 2016). The transport of particles in confined geometries is governed by complex phenomena, and the relevant interactions are sketched in Fig. 1.

A microparticle suspended in a flow experiences numerous forces, i.e., inertia, attraction, repulsion, buoyant weight, drag and surface forces (Herzig et al. 1970; McDowell-Boyer et al. 1986; Sharma and Yortsos 1987). Due to the presence of attractive interactions, the microparticles arrange themselves in certain structures/patterns which are called aggregates. These aggregates act as precursor for the formation of even larger aggregates by collision and adhesion, this phenomena is called agglomeration. Depending on the balance between particle–fluid interactions and the inter-particle interactions fragmentation can also take place, i.e., large aggregate structures break down into small aggregates or even into single particles. Both fragmentation and agglomeration often occur simultaneously, but an imbalance between these mechanisms may lead to channel clogging (Henry et al. 2013).

A generic description of the clogging process is given by the interplay between channel constriction and channel bridging. The microparticles in the flow will continuously deposit on the channel walls (particle–surface interaction), and will therefore constrict the free cross-section available for fluid flow. In addition, the particle–particle interaction can lead to the formation of an arch of particles, which then bridges across the width of the constricted channel. And even without wall deposition clogging can occur by aggregation only, as a steady increase in the agglomerate size will eventually block the channel cross-section (Goldsztein and Santamarina 2004; Sharp and Adrian 2005; Mustin and Stoeber 2010).

Early stage aggregates are primarily formed via adhesive particle–particle collisions, and these initial particle aggregates then act as a nucleus for further aggregation, and in subsequent steps several aggregates attach together to generate a single large-sized cluster (Gudipaty et al. 2011). It is observed that the region near the channel walls and in the center of the channel exhibits a higher aggregation rate, along with fragmentation from the walls (Flamm et al. 2011). The deposition of particles on the channel wall can occur as either mono or multilayers, which is influenced by several factors, such as fluid characteristics (e.g., the ionic strength), hydrodynamic conditions (e.g., Reynolds number), substrate (e.g., Zeta potential) and particle properties. In case of high flow rates, multilayer deposition is only observed when the inter-particle repulsion magnitude is low (Ramachandran and Fogler 1998, 1999; Henry et al. 2012).

Numerical computations are a promising approach to further study the effect of particle–particle interaction and wall deposition in microfluidics. The Discrete Element Method (DEM) was applied to numerically investigate aerosol particle aggregate formation and collision-induced adhesion in a periodic straight channel under laminar flow conditions (Marshall 2007, 2009). DEM computes the behavior of each particle corresponding to Newtons second law of motion, and therefore it is straightforward to include additional physics in the model, e.g., a cohesive force model and a contact model (Washino et al. 2013; Gao et al. 2014; Jasevičius et al. 2015). The results obtained by Marshall (2007, 2009) show that the adhesive properties of the particles and the flow rate have an enormous impact on the dynamics of the entire system. At large values of both flow rate and adhesive force, relatively large size aggregates are formed that deposit on the walls. This in turn affects the average velocity profile, which is disturbed due to these large aggregates adhering to the walls (Marshall 2007, 2009; Shahzad et al. 2016). A force-coupling numerical method was recently implemented to investigate microparticle aggregation in suspension flow in a three-dimensional microchannel (Agbangla et al. 2014). This method automatically considers inter-particle hydrodynamic interactions. However, due to the large computational cost the overall number of particles that can be simulated with this method is significantly lower as compared to DEM. A DLVO force has been applied to accurately model the adhesive, attractive and repulsive inter-particle interactions. Agbangla et al. concluded that inter-particle repulsive forces are the key parameter to initiate aggregate formation in the near wall region, which then will gradually block the channel.

In the current study, we applied a Discrete Element Method (DEM) and CFD–DEM coupling method to numerically simulate aggregation, agglomeration and fragmentation of rigid microparticles suspended in a Newtonian fluid flowing through a microchannel. We employed the JKR (Johnson–Kendall–Roberts) contact model to simulate particle–particle collision and adhesion in the system (Johnson et al. 1971). This contact model has recently been successfully applied to study aggregation, agglomeration and fragmentation processes in particulate systems (Marshall 2007, 2009; Shahzad et al. 2016), and to identify the cluster generation in shear-induced coagulation of microparticles flowing in water (Kroupa et al. 2014, 2015).

The present work builds on the previous publication by Shahzad et al. (2016) which was the first to implement the JKR contact model to investigate the dynamics of aggregation, agglomeration and fragmentation for micro-gel particles. To achieve a better understanding of these interaction phenomena, particularly in the entrance section of a microchannel, where the initial particle and wall contact occurs, we study the flow of solids and fluid in a non-periodic domain. In this setup, the entire microchannel is initially filled with the fluid, and based on a pre-defined solid volume fraction, particles are injected with the incoming fluid. We systematically address the aggregation and deposition behavior of the particles as a function of the applied Reynolds number and strength of the adhesive force. Furthermore, we compare simulations in two- and three-dimensional domains and we also investigate the influence of one-way (DEM) vs. two-way coupling (CFD–DEM) in microchannels.

## Mathematical model

**Fig. 2:**
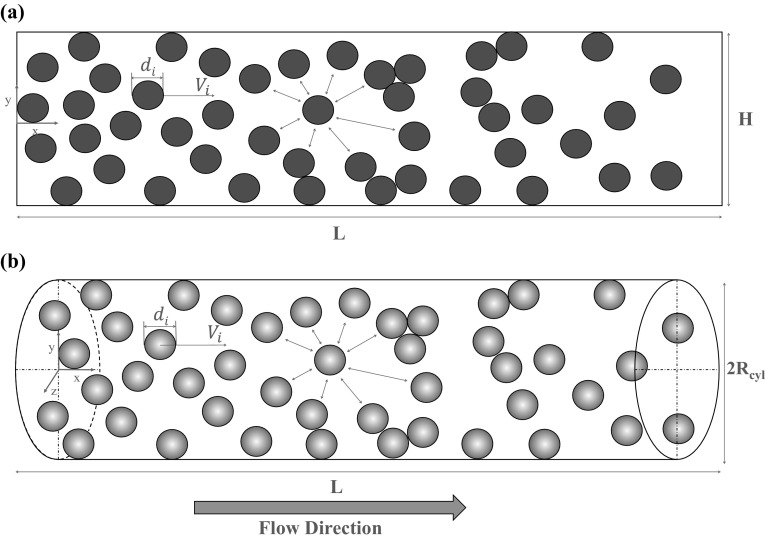
Schematic representation of the **a** 2D and **b** cylindrical (3D) microchannel

In this work, we investigate a suspension of rigid microparticles flowing through a microchannel, which is represented as a two- (2D) and three-dimensional (3D) cylindrical channel of length *L*. Figure 2 depicts a schematic overview of both computational domains.

For the 2D channel geometry (Fig. 2a), the total length of the channel is eight times the channel width *H*, hence a fully developed velocity profile is achieved in the gap. Circular rigid microparticles with a specified diameter $d_{\text {P}}$ are suspended in the fluid. A Cartesian coordinate system with the origin in the center of the microchannel is placed in the inlet section, where *x* represents the fluid flow direction. The 3D cylindrical geometry (Fig. 2b) has a total length of eight times the channel diameter $2 R_{\text {cyl}}$, which also leads to a fully developed velocity profile. Here, we consider a 3D cylindrical coordinate system with the origin at the center of the channel inlet section, where *x* also represents the fluid flow direction. In the 3D system, spherical microparticles with a specified diameter $d_{\text {P}}$ are suspended in the fluid.

In the one-way coupling approach, the flow field is assumed to be unaffected by the particle interactions. However, in the two-way coupling approach, both flow field and particle dynamics are computed simultaneously. Initially, a steady flow field is achieved in the microchannel, upon which particles are randomly injected on the inlet patch by defining the ‘Particle Adding Frequency’ $f_{\text {add}}$ (see Table 1) in such a way that the overall volume fraction remains constant throughout the entire channel. In addition, the velocity of the injected particles is set equal to the local fluid velocity. We impose non-periodic boundary conditions, as soon as a particle crosses the outlet patch it is instantaneously removed from the computation. The developed DEM code used here has been validated in a previous work (Shahzad et al. 2016) by comparing reported results of a particulate aerosol flow (Marshall 2007).

**Table 1:** Geometric details, fluid and particle properties, and derived parameters used in the 2D and 3D simulations

Parameter	Value
Particle diameter	$d_{\text {p}}$= 10 $\upmu$m
Microchannel length	*L* = 2 mm
Channel width (2D)	*H* = 0.25 mm
Channel radius (3D)	$R_{\text {cyl}}$ = 0.125 mm
Volume fraction (2D)	$\phi _{\text {2D}} = 0.1$
Volume fraction (3D)	$\phi _{\text {3D}} = 0.05$
Particle density	$\rho _{\text {p}}$ = 2600 kg/m$^{3}$
Fluid density	$\rho _{\text {f}}$ = 1000 kg/m$^{3}$
Fluid viscosity	$\mu$ = 0.001 kg/(m s)
Elastic modulus	*E* = 1 GPa
Particle adding frequency	$f_{\text {add}} = \frac{6\phi _{\text {2D/3D}} U_{\text {f}} R_{\text {cyl}}^2}{d_{\text {P}}^{3}}$
Total number of particles	$\bar{N}_{\text {tot}}(t) = \frac{{N}_{\text {tot}}(t)}{N_{\text {tot}}(t\rightarrow \infty ,\phi =0)}$
Aggregate percentage	$\% \text {Aggregate}_{i} = \frac{C_{i,s}}{\sum _{s} C_{i,s}}$
Fluid velocity $(Re = 0.75, 1.0, 1.5)$	$U_{\text {f}} = 2.91, 3.8889, 5.8333$ mm/s
Time step size $(Re = 0.75, 1.0, 1.5)$	$\Delta t = 1.16 {\text {e}}^{-6}, 1.56 {\text {e}}^{-6}, 2.36 {\text {e}}^{-6}$ s

### Discrete element method (DEM): one-way coupling approach

In the implemented one-way coupling approach, the microparticles are transported by the fluid while experiencing collisions with each other, however, the flow field of the fluid carrying the suspended microparticles is not modifiable (Marshall 2007). The overall dynamics of the microparticles is simulated by employing the Discrete Element Method (DEM), i.e., we employ a Lagrangian model to calculate the trajectories of the particles by solving the equations of motion for translational and angular velocities, respectively (Cundall and Strack 1979; Landry et al. 2003; You et al. 2004),

1 $$\begin{aligned} m\frac{{\text {d}}\varvec{v}}{{\text {d}}t}= \varvec{F}_{\text {D}} + \varvec{F}_{\text {A}}, \end{aligned}$$

2 $$\begin{aligned} I\frac{{\text {d}}\varvec{\Omega }}{{\text {d}}t}= \varvec{M}_{\text {D}} + \varvec{M}_{\text {A}}, \end{aligned}$$

where, *m* and *I* represent the particle mass and momentum of inertia, respectively, $\varvec{v}$ and $\varvec{\Omega }$ are the particle translation and angular velocities, respectively. We assume that no additional forces (or torques) arise due to sliding and twisting motions of particles interacting with each other. These assumptions can be justified as the Reynolds and Stokes numbers imposed in the simulations are rather small (Shahzad et al. 2016). Consequently, the only forces experienced by the particles are the fluid drag force $\varvec{F}_{\text {D}}$, and $\varvec{F}_{\text {A}}$ the sum of the van der Waals adhesive and the elastic collision forces. The fluid torque $\varvec{M}_{\text {D}}$ and the sum of the van der Waals adhesion and elastic collisions torques $\varvec{M}_{\text {A}}$ are not considered, Eqs. (1) and (2) are decoupled as the particle translational motion is unaffected by its angular rotation (Shahzad et al. 2016). We also assume a ‘freeze’ boundary condition, i.e., when a particle gets in contact with the channel wall it will be permanently adhered. In the one-way coupling approach, the fluid–particle interaction close to the wall is minimal, and therefore resuspension of particles is not expected. However, for rather low adhesion forces, very few particles are expected to attach to the walls, but nevertheless, the flowing aggregate dynamics remain unaffected.

The force terms used in Eq. (1) are discussed in Sect. S1 of the Supporting Information, and for further information on the model implementation we refer to Shahzad et al. (2016).

### CFD–DEM: two-way coupling approach

The CFD–DEM coupling approach is adopted to study aggregate formation and clogging in the 3D cylindrical channel while incorporating particle–particle, particle–wall, and particle–fluid interactions. In this coupled approach, the continuous liquid phase is represented by solving the volume-averaged Navier–Stokes equations using the open-source toolbox OpenFOAM, while employing DEM to model the particulate phase using the open-source toolbox LIGGGHTS. The open source CFDEM-coupling (Kloss et al. 2012) is used for data exchange between the two solvers at regular intervals; generally several DEM time steps are coupled with a single CFD time step. In the following Sects. 2.2.1 and 2.2.2, the equations and parameters used in the CFD–DEM model are briefly discussed.

#### DEM for solid phase

As outlined above, DEM is based on solving Newton’s second law to compute translational and rotational velocity and position of each particle in time. All the equations in this section are computed using LIGGGHTS implementing Zhou et al. (2010) notations:

3 $$\begin{aligned} m \frac{{\text {d}}\varvec{v}}{{\text {dt}}}= \varvec{F}_{\text {n}} +\varvec{F}_{\text {t}} + \varvec{F}_{\text {pf}} + \varvec{F}_{\text {g}}, \end{aligned}$$

4 $$\begin{aligned} I \frac{{\text {d}}\varvec{\Omega }}{{\text {d}}t}= \varvec{M}_{\text {t}} +\varvec{M}_{\text {r}}, \end{aligned}$$

where *m* and *I* are the particle mass and momentum of inertia, respectively, $\varvec{v}$ and $\varvec{\Omega }$ are the translational and angular velocities of the particle, respectively, $\varvec{F}_{\text {n}}$ and $\varvec{F}_{\text {t}}$ are the normal and tangential contact force between particles *i* and *j*, respectively, $\varvec{F}_{\text {pf}}$ is the particle–fluid interaction force, $\varvec{F}_{\text {g}}$ is the gravitational force, and $\varvec{M}_{\text {t}}$ and $\varvec{M}_{\text {r}}$ are the tangential and rolling friction momentum acting on *i* and *j* particles, respectively. In this work, we neglect gravitational and non-contact forces (i.e., electrostatic interaction or van der Waals forces), as the hydrodynamic and contact forces are orders of magnitude larger. We used the Johnson–Kendall–Roberts (JKR) contact model (Johnson et al. 1971) as described in Sect. S1.2, which was also validated for hard gel microparticles in our previous work (Shahzad et al. 2016). Table S1 in the Supporting Information summarizes the equations to calculate the forces and parameters in the DEM part of the CFD–DEM coupling.

#### CFD for liquid phase

Once all the force and torque terms are numerically solved in DEM, i.e., the trajectories and velocities for each particle are known, the CFD computation is performed to solve the motion of an incompressible fluid in the presence of the solid phase. For this, a modified set of the Naiver–Stokes equations based on the local volume average method are used (Gidaspow 1994; Zhou et al. 2010). In the present work, we use ‘Model type A’ that computes a shared pressure drop between the solid and fluid phase, in contrast to ‘Model type B’ which considers only the pressure drop for the fluid phase (Blais et al. 2016).

5 $$\begin{aligned}&\frac{\partial \epsilon _{\text {f}}}{\partial t} + \nabla \cdot \left( \epsilon _{\text {f}} \varvec{u}_{\text {f}}\right) = 0, \end{aligned}$$

6 $$\begin{aligned}&\frac{\partial \left( \rho _{\text {f}}\epsilon _{\text {f}} \varvec{u}_{\text {f}}\right) }{\partial t} + \nabla \cdot \left( \rho _{\text {f}}\epsilon _{\text {f}} \varvec{u}_{\text {f}}\varvec{u}_{\text {f}}\right) = - \epsilon _{\text {f}} \nabla p - \varvec{F}^A_{\text {pf}} + \nabla \cdot \tau + \rho _{\text {f}}\epsilon _{\text {f}} \mathbf g , \end{aligned}$$

7 $$\begin{aligned}&\varvec{\tau } = \epsilon _{\text {f}}\mu \left( \left( \nabla \varvec{u}\right) + \left( \nabla \varvec{u}\right) ^{\text {T}} - \frac{2}{3} \left( \nabla \cdot \varvec{u}\right) \delta _{k} \right) , \end{aligned}$$

8 $$\begin{aligned}&\varvec{F}^A_{\text {pf}} = - \varvec{K}_{\text {pf}}(\varvec{u}_{\text {p}} - \varvec{u}),\end{aligned}$$

9 $$\begin{aligned}&\text {K}_{\text {pf}} = \frac{|\varvec{F}_{\text {pf}}|}{|\varvec{v} - \varvec{u}_{\text {f}}|}, \end{aligned}$$

10 $$\begin{aligned}&\varvec{F}_{{\text {pf}},i} = \varvec{f}_{{\text {d}},i} + \varvec{f}_{\nabla p,i} + \varvec{f}_{\nabla \tau ,i}, \end{aligned}$$

where $\varvec{u}_{\text {f}}$ and *p* are the fluid velocity and pressure terms, respectively, $\epsilon _{\text {f}}$, $\tau$ and $\Delta V$ are the fluid volume fraction in each cell, fluid viscous stress tensor, and the volume of the computational cell, respectively. $\text {K}_{\text {pf}}$ is a scalar term used to scale the magnitude of the momentum exchange force, $\varvec{F}^A_{\text {pf}}$ is the volumetric particle–fluid interaction force and $\varvec{F}_{{\text {pf}},i}$ is the particle–fluid interaction force, $\varvec{f}_{{\text {d}},i}$ represents drag, $\varvec{f}_{\nabla p,i}$ the pressure gradient and $\varvec{f}_{\nabla \tau ,i}$ the viscous stress (or shear stress) on each particle, respectively. Furthermore, we used an unresolved CFD–DEM coupling approach, which is only feasible when the particle size is smaller than the cells of the computational grid.

The CFD–DEM coupling strategy is detailed in Sect. S3 in the Supporting Information. For the discretization of the 3D case we generated a structured mesh consisting of 84,000 cells. The equations are solved using the *cfdemSolverPiso* solver, which is based on the PISO algorithm, where two PISO loops are performed for each time step and within each PISO loop 2 non-orthogonal correction loops are applied.

## Results and discussion

From the geometric details, fluid and particle properties (see Table 1), five dimensionless groups can be defined for this problem. These are the confinement ratios (based on geometry):

$$\begin{aligned} \beta = \frac{d_{\text {p}}}{2 R_{{\text {cyl}}}}, \end{aligned}$$

the density ratio (based on physical properties)

$$\begin{aligned} \rho _{\text {r}} = \frac{\rho _{\text {p}}}{\rho _{\text {f}}}, \end{aligned}$$

the elasticity parameter (based on physical and flow properties)

$$\begin{aligned} \lambda = \frac{E}{\rho _{\text {p}} U_{\text {f}}^2}, \end{aligned}$$

the fluid Reynolds number (based on geometry, physical and flow properties)

$$\begin{aligned} {Re} = \frac{\rho _{\text {f}} R_{{\text {cyl}}} U_{\text {f}}}{\mu }, \end{aligned}$$

and the adhesion parameter (based on geometry, physical and flow properties)

$$\begin{aligned} \phi = \frac{\gamma }{\rho _{\text {p}} U_{\text {f}}^2 R}. \end{aligned}$$

In the present study, we fixed the geometry and particle size, and systematically varied the fluid velocity $U_{\text {f}}$ and the surface energy $\gamma$ of the particles to characterize their effect on particle–fluid, particle–particle, and particle–wall interactions. The resulting Reynolds numbers, elasticity and adhesion parameters are listed in Table 2.

**Table 2:** Overview of all studied cases

Cases	Model	*Re*	$\phi$	$\lambda$	$t_{\text {2D}}$	$t_{\text {3D}}$
A.1	DEM	0.75	15	$2.9387 \times 10^{8}$	116	50
A.2	DEM	0.75	150	$2.9387 \times 10^{8}$	116	50
A.3	DEM	0.75	1500	$2.9387 \times 10^{8}$	116	50
A.4	DEM	0.75	15,000	$2.9387 \times 10^{8}$	116	50
B.1	DEM	1.0	15	$1.65306 \times 10^{8}$	155	−
B.2	DEM	1.0	150	$1.65306 \times 10^{8}$	155	−
B.3	DEM	1.0	1500	$1.65306 \times 10^{8}$	155	−
B.4	DEM	1.0	15,000	$1.65306 \times 10^{8}$	155	−
C.1	DEM	1.5	15	$7.34695 \times 10^{7}$	235	−
C.2	DEM	1.5	150	$7.34695 \times 10^{7}$	235	−
C.3	DEM	1.5	1500	$7.34695 \times 10^{7}$	235	−
C.4	DEM	1.5	15,000	$7.34695 \times 10^{7}$	235	−
D.1	CFD–DEM	0.75	15,000	$2.9387 \times 10^{8}$	−	50
D.2	CFD–DEM	1.5	15,000	$7.34695 \times 10^{7}$	−	50

The last two columns represent the final dimensionless time for the 2D and 3D microchannels

In the following, the complexity of the simulations is increased incrementally by characterizing particle aggregation and clogging in a 2D microchannel (one-way coupling), 3D microchannel (one-way coupling), and finally a 3D microchannel using two-way coupling.

### One-way coupling approach in 2D microchannel

**Fig. 3:**
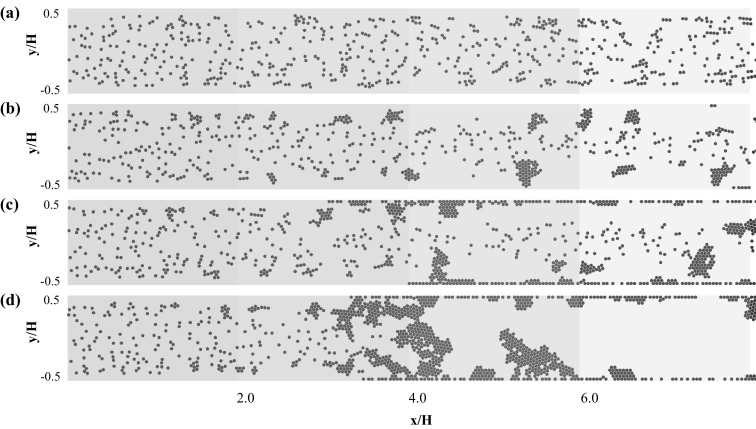
Particle distribution in the 2D channel for Reynolds number ${Re}=0.75$ and adhesion parameters **a** $\phi =15$, **b** $\phi =150$, **c** $\phi =1500$, and **d** $\phi =15{,}000$. The particle distribution is plotted at the end of the simulations ($t_{\text {2D}}=116$)

**Fig. 4:**
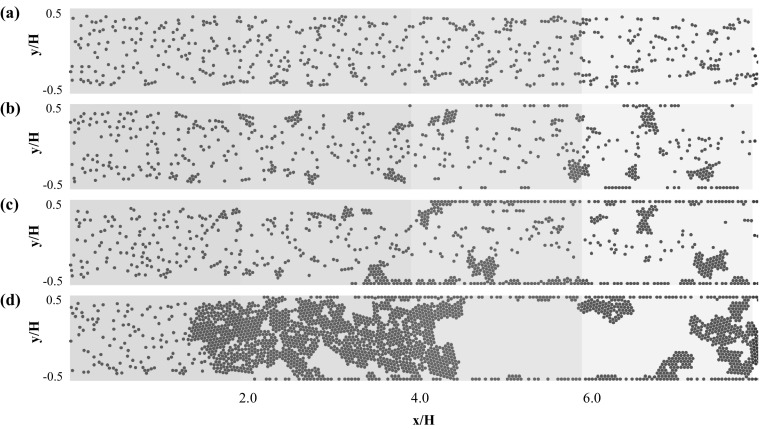
Particle distribution in the 2D channel for Reynolds number ${Re}=1.0$ and adhesion parameters **a** $\phi =15$, **b** $\phi =150$, **c** $\phi =1500$, and **d** $\phi =15{,}000$. The particle distribution is plotted at the end of the simulations ($t_{\text {2D}}=155$)

**Fig. 5:**
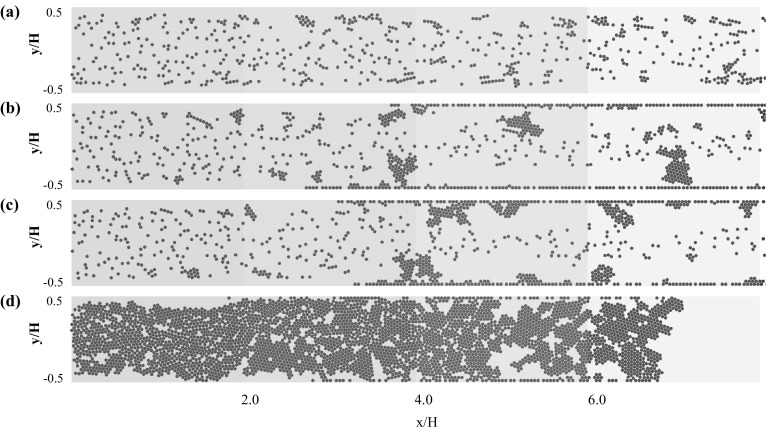
Particle distribution in the 2D channel for Reynolds number ${Re}=1.5$ and adhesion parameters **a** $\phi =15$, **b** $\phi =150$, **c** $\phi =1500$, and **d** $\phi =15{,}000$. The particle distribution is plotted at the end of the simulations ($t_{\text {2D}}=235$)

Figures 3, 4, and 5 depict the rigid microparticle distribution in the two-dimensional rectangular microchannel for Reynolds numbers ${Re}=$ 0.75, 1.0, 1.5, respectively. Each figure contains four snapshots taken at the final simulation time and for different values of the adhesion parameter $\phi$ = 15, 150, 1500, and 15,000 in ascending order from top to bottom. For all the cases, the dimensionless final simulation time $t_{\text {2D}}=\frac{U_{\text {f}} t}{H}$ is listed in Table 2. All simulations are performed sufficiently long to achieve steady state, which is confirmed by monitoring the aggregation statistics. The only exception is case C.4 (Fig. 5d), as the channel clogged without ever reaching steady state.

It is generally observed that an increase in the adhesion parameter generates larger aggregates at a fixed Reynolds number. Considering the lowest Reynolds number $Re=0.75$ and adhesion parameter $\phi =15$ (Fig. 3a), only small size aggregates are present which are mostly formed of 2–3 particles, with the remaining suspended particles leaving the flow domain without interacting with each other or the walls. Gradual increase in the adhesion parameter results in an increased formation of aggregates, especially in the near wall region. This observation is in line with the process of orthokinetic aggregation, in which the aggregation rate scales with the local shear rate, which is highest in the near region for the here considered developed laminar flow (Elimelech et al. 2013). These initially formed aggregates increase in size as they pass through the channel and some wall deposition is observed (Fig. 3c, d). The first monolayer of particles will permanently adhere to the channel walls according to our implemented freeze boundary condition. However, the second layer can attach and detach from this monolayer depending on the balance between adhesive and fluid-induced friction forces.

When increasing the Reynolds number to $Re=1.0$, aggregate formation in the near wall region is already observed at the lowest adhesion parameter (Fig. 4a). Increasing the adhesion parameter at this particular Reynolds number promotes increased aggregate formation and wall deposition. However, the detachment of aggregates induced by particle–fluid interaction is also observed. At the highest value of the adhesion parameter $\phi =15{,}000$, the formation of dendritic structures attached to the channel wall is observed. While protruding into the flow, these structures stay attached due to the large adhesion force. Free flowing aggregates can collide with these structures, and upon impact either attach and merge with them, or result in breakage and detachment of the dendritic structure.

Increasing the Reynolds number further to the highest considered value of $Re=1.5$, an increase in the deposition and the aggregation dynamics is observed (Fig. 5). A monolayer of particles deposited on the walls is observed for adhesion parameters exceeding $\phi =150$, and furthermore, the particle aggregates formed in the near wall region are larger in size compared to the lower Reynolds number cases. This indicates an increased probability of particle–particle collision when increasing the fluid flow rate (Elimelech et al. 2013). As observed earlier, when increasing the adhesion parameter value the number of aggregates and their sizes increase as well, which in the case of the largest adhesion parameter of $\phi =15{,}000$ leads to increased merging of free flowing and wall attached dendritic structures (Fig. 5d). These structures bridge the entire channel height and drastically reduce the effective cross-sectional area of the microchannel, thus indicating the onset of channel clogging.

To study this particle aggregation dynamics in more detail, the entire flow channel is subdivided into four equal volumes, which are highlighted by different colors in Figs. 3, 4, and 5: $x/H=0.0$ represents the microchannel inlet and $x/H=8.0$ its outlet, with intermediate intersections at $x/H=2.0$, $x/H=4.0$, and $x/H=6.0$. Whenever the center of mass of an aggregate crosses these intersections, its size, defined as the number of particles forming it, is recorded. This information is then used to obtain the spatial distribution of the percentage of aggregates (denoted as $\% \text {Aggregate}$) of a certain size according to

11 $$\begin{aligned} \% \text {Aggregate}_{i} = \frac{C_{i,s}}{\sum _{s} C_{i,s}} , \end{aligned}$$

where $C_{i,s}$ is the number of aggregates of size *s* (with $s\ge 1$) crossing the intersection *i*, and the denominator represents the total number of aggregates crossing intersection *i*.

**Fig. 6:**
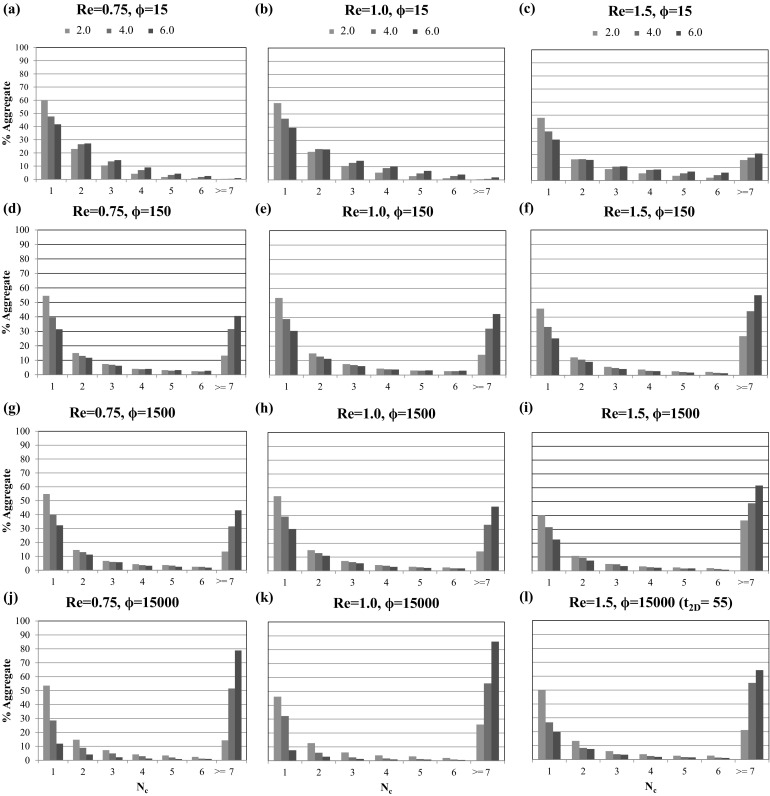
Percentage of aggregates $\% \text {Aggregate}$ consisting of $N_{\text {c}}$ particles per aggregate at the three intersections $x/H=2.0$ (green), $x/H=4.0$ (blue), and $x/H=6.0$ (red) for Reynolds numbers ${Re}=0.75$ (left column), ${Re}=1.0$ (middle column), ${Re}=1.5$ (right column) and adhesion parameters $\phi =15$ (first row), $\phi =150$ (second row), $\phi =1500$ (third row), $\phi =15{,}000$ (fourth row). (Color figure online)

Figure 6 depicts the percentage of aggregates $\% \text {Aggregate}$ consisting of $N_{\text {c}}$ particles per aggregate at the three intersections for all considered 2D microchannel cases. Each figure panel contains three color bars, which represent the three intersections at $x/H=2.0$ (green bar), $x/H=4.0$ (blue bar), and $x/H=6.0$ (red bar). The individual figure panel columns represent fixed Reynolds numbers (increasing from left to right), while the rows correspond to fixed values of the adhesion parameter (increasing from top to bottom).

These statistics highlight that for all simulated cases the number of non-aggregated single particles is decreasing in the streamwise direction, due to the aggregation processes visually observed in Figs. 3, 4, and 5. For the smallest value of the adhesion parameter ($\phi =15$) and Reynolds numbers of $Re=0.75$ and $Re=1.0$ the aggregation process is still relatively slow, with the majority of aggregates being comprised of 1–6 particles per aggregate. For all other cases, a substantial fraction of the formed clusters consist of seven or more particles ($N_{\text {c}} \ge 7$), and the percentages of aggregates in this size range increase with increasing Reynolds number and adhesion parameter. It is also worth noting that the formation of these large clusters appears to be primarily taking place in the second half of the microchannel, which can be explained by the merging of smaller free flowing aggregates.

The accumulation of particles inside the microchannel is calculated by comparing the temporal evolution of the total number of particles present in the channel ${N}_{\text {tot}}(t)$ with the steady-state value of the total number of particles present in the channel in the absence of adhesive forces $N_{\text {tot}}(t\rightarrow \infty ,\phi =0)$ according to

12 $$\begin{aligned} \bar{N}_{\text {tot}}(t) = \frac{{N}_{\text {tot}}(t)}{N_{\text {tot}}(t\rightarrow \infty ,\phi =0)}. \end{aligned}$$

**Fig. 7:**
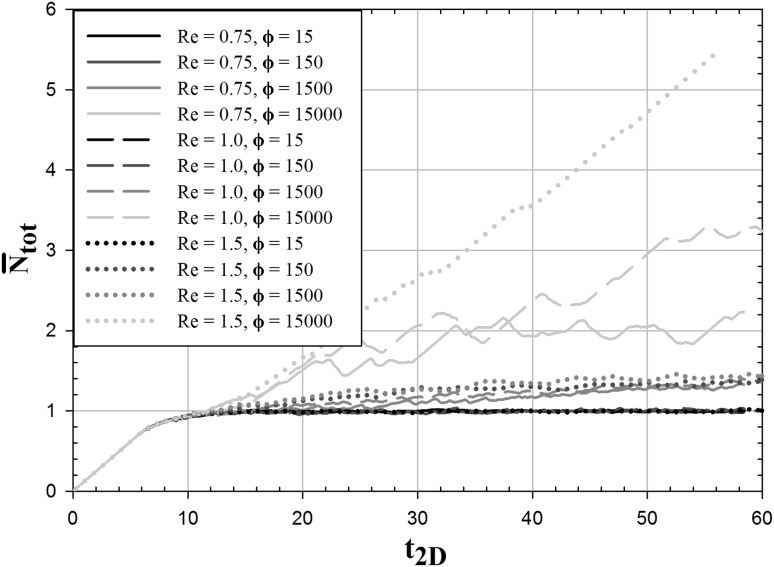
Temporal evolution with respect to the dimensionless simulation time $t_{\text {2D}}$ of the number of particles present in the 2D microchannel normalized with the steady-state number for ${Re}=0.75$, ${Re}=1.0$, ${Re}=1.5$ and $\phi =15$, $\phi =150$, $\phi =1500$, $\phi =15{,}000$

The temporal evolution of this dimensionless number of accumulated particles is depicted in Fig. 7 for all the simulated cases. Up to a dimensionless simulation time of $t_{\text {2D}}=10$, all curves overlap and approach the steady-state value of $\bar{N}_{\text {tot}}=1$. This transient behavior is caused by the simulation procedure, the microchannel is initially filled with the fluid phase only, and the particles are introduced via the inlet patch according to a specified particle adding frequency.

Hardly any particle accumulation is observed for all flow rates for the lowest values of the adhesion parameter $\phi =15$ and $\phi =150$ and the two lowest values of the Reynolds number ($Re=0.75$; $Re=1.0$) as $\bar{N}_{\text {tot}}\approx 1$ over time for these cases. As depicted in Fig. 6 aggregate formation is also occurring for these five cases, but all formed aggregates are free flowing and exit the microchannel without deposition on the channel walls. Upon a further increase of the flow rate ($Re> 1.0$) and the adhesion parameter ($\phi>150$) particle accumulation is observed, which is increasing over time and with the magnitude of the rate of increase depending on the specific values of Re and $\phi$. As expected, the largest particle accumulation is found for the largest considered adhesion parameter of $\phi =15{,}000$. For a Reynolds number of $Re=1.5$, the largest rate of increase in the number of particles accumulated in the microchannel is observed, which indicates the onset of channel clogging as shown in Fig. 5d. The oscillations observed in all the curves are associated with the continuous generation of aggregates and their exit from the channel domain. The amplitude value from those oscillations provide us generic information about aggregate sizes.

**Fig. 8:**
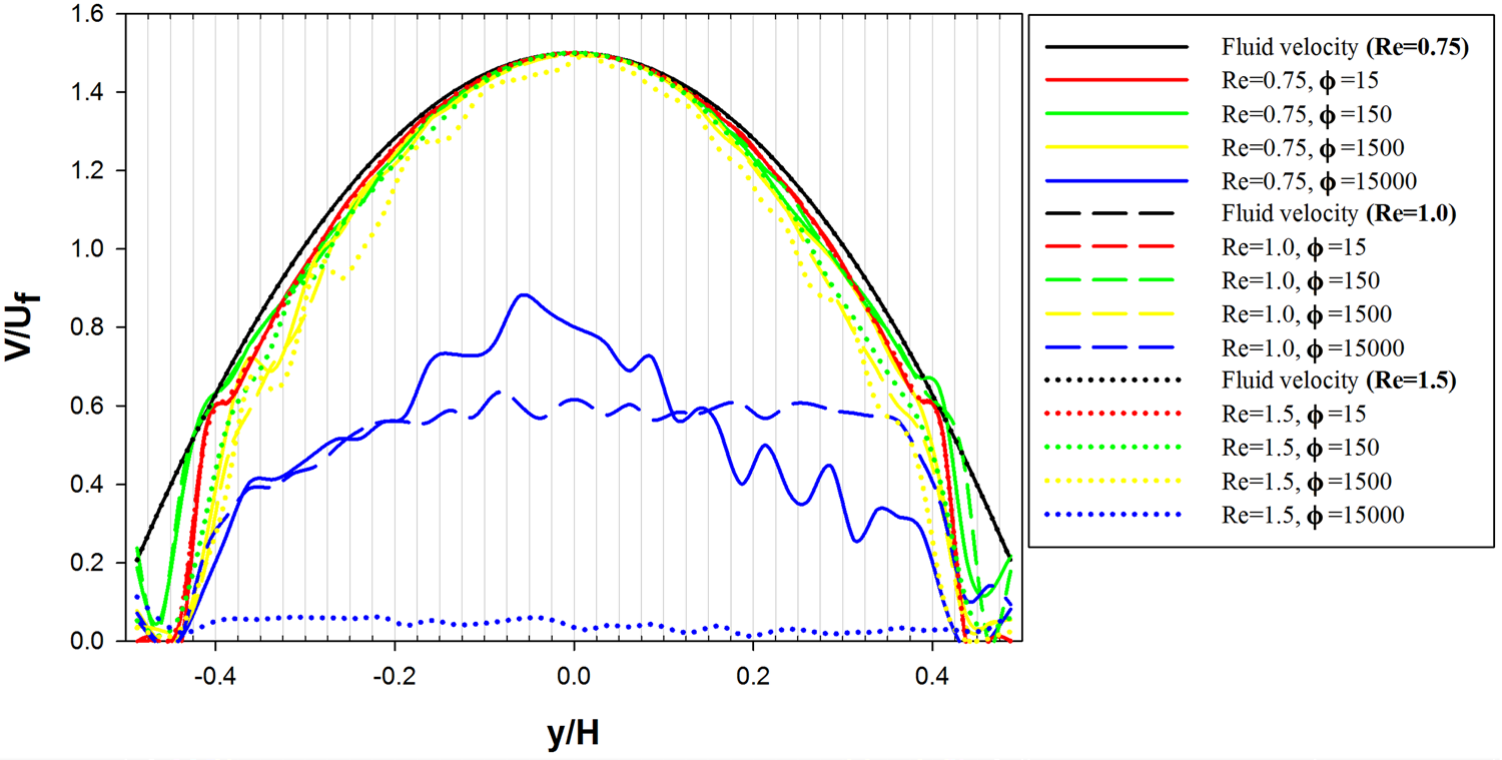
Averaged particle and streamwise fluid velocity profiles for the 2D microchannel at ${Re}=0.75$, ${Re}=1.0$, ${Re}=1.5$ and $\phi =15$, $\phi =150$, $\phi =1500$, $\phi =15{,}000$

The averaged particle and streamwise fluid velocity for the 2D microchannel cases are shown in Fig. 8. To obtain the averaged velocities, the microchannel is subdivided into regions with a dimensionless width of $y/H=0.025$ extending along the entire channel length *L* in flow direction (these regions are indicated in Fig. 8 by vertical lines). The shown particle and fluid velocity profile corresponds to the average velocity of all particles, respectively fluid elements, in each region.

As one-way coupling is used for the 2D microchannel simulations, the streamwise fluid velocity profile equals the profile for fully developed laminar flow. No significant deviation between the fluid and particle velocity is observed in the central region of the microchannel ($-0.25<y/H<0.25$) for adhesion parameter values $\phi \le 1500$ and all considered flow rates. In the near wall regions ($y/H<-0.4$ and $y/H>0.4$), the particle velocity is smaller compared to the fluid velocity, which is caused by aggregate formation and the formation of dendritic structures attached to the microchannel walls. For the largest considered value of the adhesion parameter $\phi =15{,}000$, a significant decrease of the particle velocity relative to the fluid velocity along the entire microchannel width *H* is found. This decrease in particle velocity is enhanced with increasing Reynolds number, which is caused by increased wall attachment and merging of free flowing with wall attached aggregates. For $Re=1.5$, the particle velocity approximates zero in the entire channel width which finally leads to clogging. These changes in the particle velocity profiles underline the observations in Figs. 3, 4, and 5. The initial aggregate formation occurs in the near wall region of the microchannel, and developing further into wall deposition and subsequent channel bridging by the growth and merging of aggregates.

### One-way and two-way coupling approaches in a cylindrical 3D microchannel

**Fig. 9:**
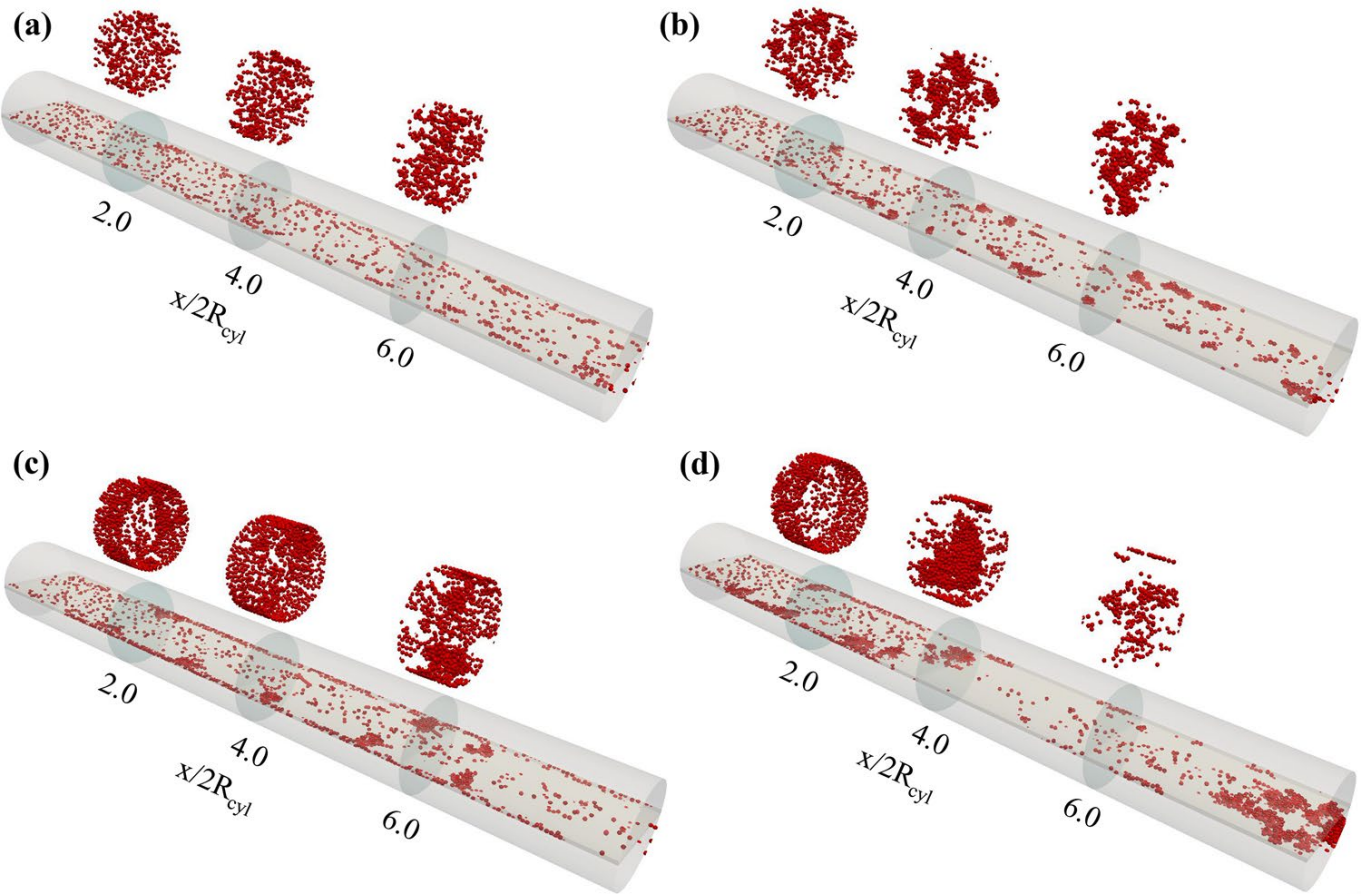
One-way coupling approach: particle distribution in the 3D channel for Reynolds number ${Re}=0.75$ and adhesion parameters **a** $\phi =15$, **b** $\phi =150$, **c** $\phi =1500$, and **d** $\phi =15{,}000$. The particle distribution is plotted at the end of the simulations ($t_{\text {3D}}=50$)

In a next step, the particle aggregation is investigated in a cylindrical 3D microchannel. Figure 9 depicts the particle distribution for a Reynolds number of $Re=0.75$ and the four different values of the adhesion parameter $\phi$ = 15, 150, 1500, and 15,000, these simulations are performed using the one-way coupling approach.

**Fig. 10:**
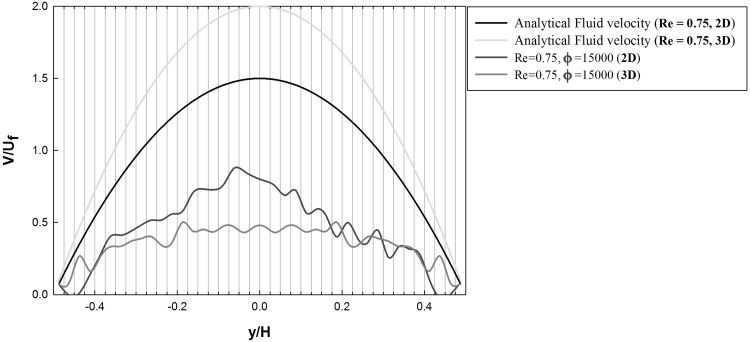
Averaged particle and streamwise fluid velocity profiles for the 2D and 3D microchannels at ${Re}=0.75$ and $\phi =15{,}000$

When comparing with the corresponding 2D microchannel results (Fig. 3), it is observed that the aggregates formed in the 3D case consist of more particles. Furthermore, the monolayer of particles attached to the wall is formed at identical adhesion parameter values ($\phi =1500$ and $\phi =15{,}000$), but the deposition process starts earlier in the 3D channel. The averaged particle and streamwise fluid velocity profiles for the 2D and 3D microchannel are compared for $Re=0.75$ and $\phi =15{,}000$ in Fig. 10.

**Fig. 11:**
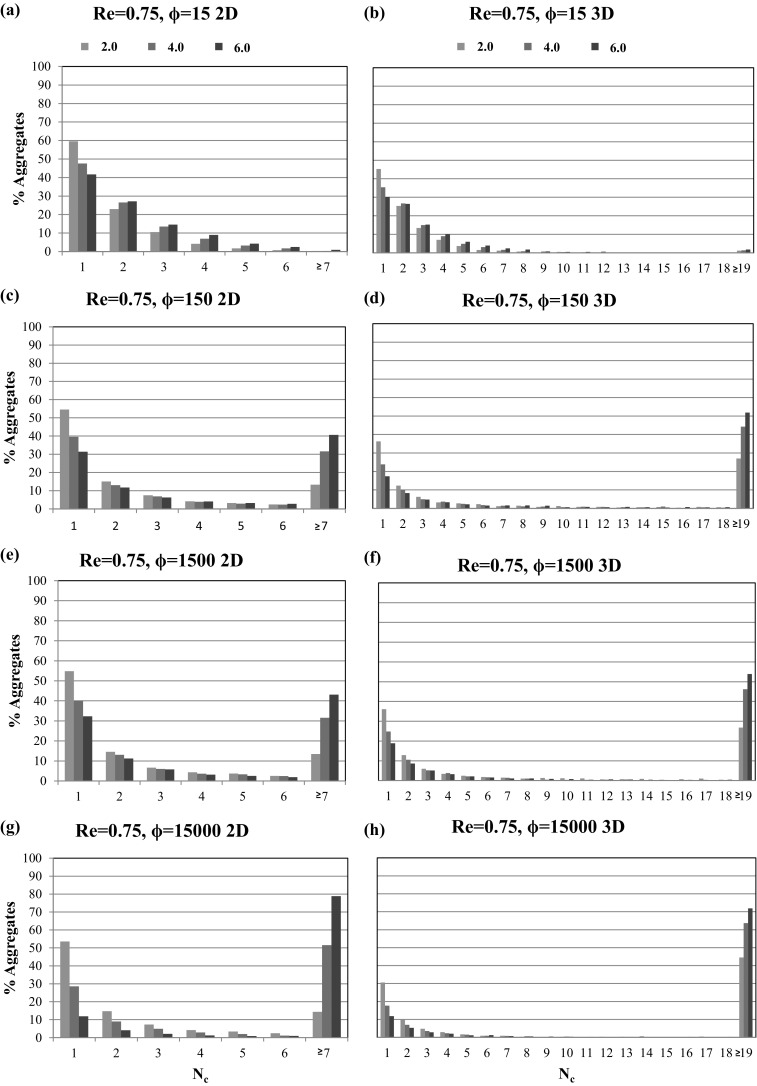
Comparison of the percentages of aggregates $\% \text {Aggregate}$ consisting of $N_{\text {c}}$ particles per aggregate at the three intersections $x/H=x/2R_{{\text {cyl}}}=2.0$ (green), $x/H=x/2R_{{\text {cyl}}}=4.0$ (blue), and $x/H=x/2R_{{\text {cyl}}}=6.0$ (red) in the 2D (left column) and 3D (right column) channel for a Reynolds number of ${Re}=0.75$ and adhesion parameters $\phi =15$ (first row), $\phi =150$ (second row), $\phi =1500$ (third row), $\phi =15{,}000$ (fourth row). (Color figure online)

While the overall averaged particle velocities are similar between the 2D and the 3D case, the relative deviation to the streamwise fluid velocity profile is more pronounced for the 3D case. This behavior is caused by enhanced aggregation dynamics, and therefore, larger aggregate sizes in the 3D microchannel, which can be quantified by comparing the percentages of aggregates $\% \text {Aggregate}$ consisting of $N_{\text {c}}$ particles per aggregate at the three intersections $x/H=x/2R_{{\text {cyl}}}=2.0, 4.0,$ and 6.0, depicted in Fig. 11.

A close resemblance in particle aggregation dynamics is found, with the exception that the aggregates formed in the 3D channel are larger ($N_{\text {c}}\ge 19$), due to the fact that the particles have an increased cross-section and volume available to arrange themselves into 3D aggregate structures. Correspondingly, the influence of both the Reynolds number and the adhesion parameter values on the percentage of aggregates is more prominent in the 3D case. At the largest considered adhesion parameter $\phi =15{,}000$, more than $70\%$ large size aggregates are formed at the streamwise position $x/H=x/2R_{{\text {cyl}}}=6.0$ as shown in Fig. 11g, h. Furthermore, in the 3D cases, very few intermediate size aggregates ($10<N_{\text {c}}<18$) are observed, which additionally highlight the enhanced aggregation dynamics in 3D.

**Fig. 12:**
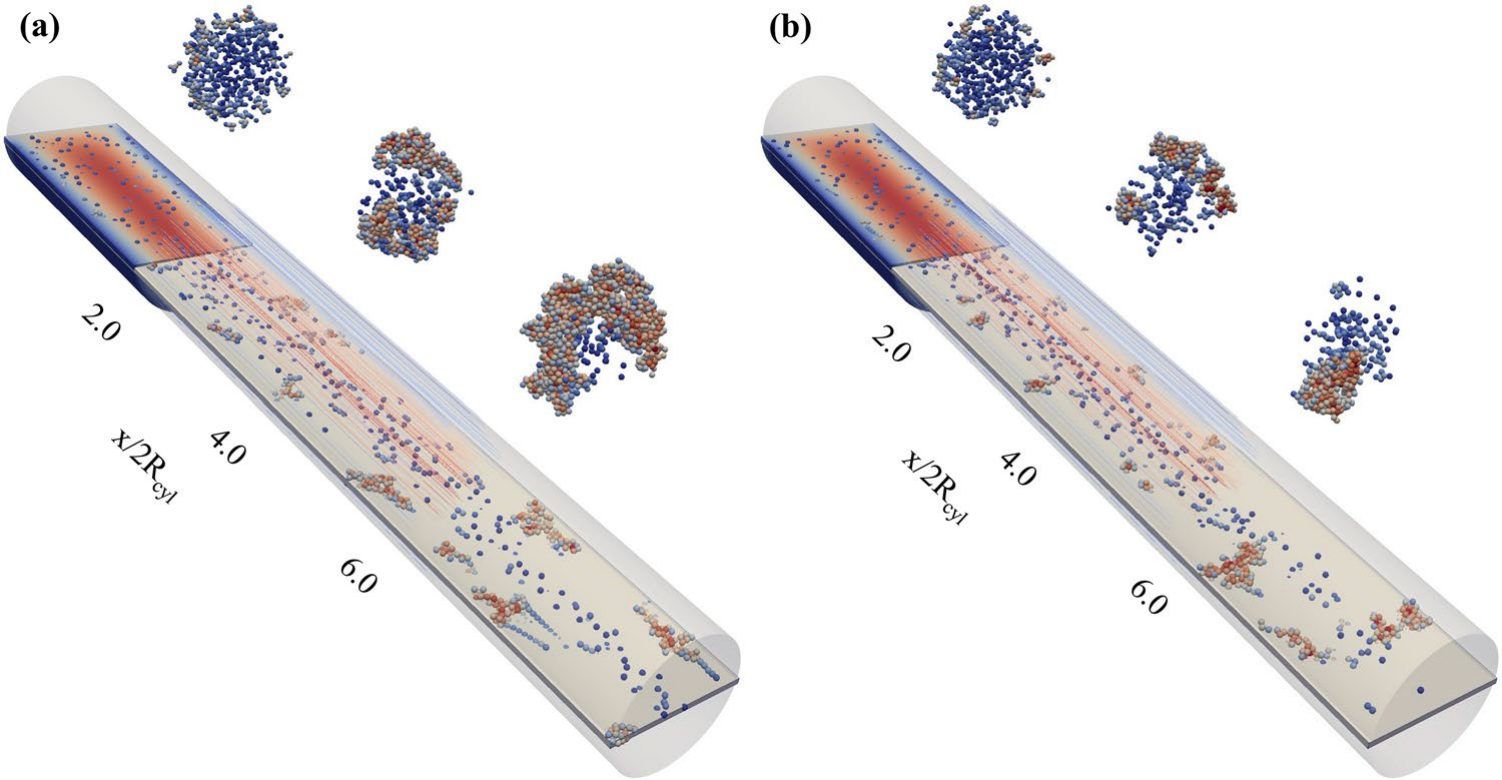
Two-way coupling approach: Particle distribution in the 3D channel for Reynolds numbers of **a** ${Re}=0.75$ and **b** ${Re}=1.5$ with an adhesion parameter of $\phi =15{,}000$. The particle distribution is plotted at the end of the simulations ($t_{\text {3D}}=50$)

Advancing to the two-way coupling approach, we are simulating the particle–fluid, particle–particle, and particle–wall interactions for Reynolds numbers $Re=0.75$ and $Re=1.5$ for an adhesion parameter of $\phi =15{,}000$. The corresponding particle distributions in the 3D channel are depicted in Fig. 12.

**Fig. 13:**
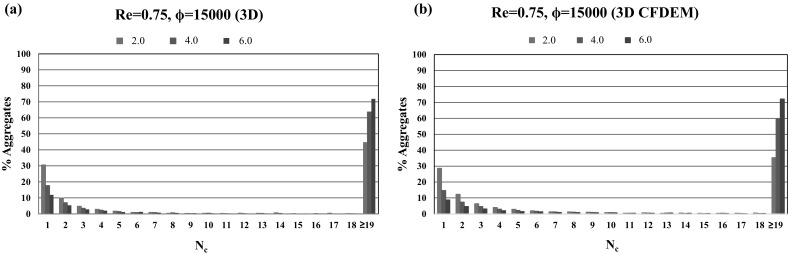
Comparison of the percentages of aggregates $\% \text {Aggregate}$ consisting of $N_{\text {c}}$ particles per aggregate at the three intersections $x/2R_{{\text {cyl}}}=2.0$ (green), $x/2R_{{\text {cyl}}}=4.0$ (blue), and $x/2R_{{\text {cyl}}}=6.0$ (red) for the **a** one-way and **b** two-way coupled simulations for a Reynolds number of ${Re}=0.75$ and adhesion parameter $\phi =15{,}000$. (Color figure online)

Comparing the one- and two-way coupling results for a Reynolds number of ${Re}=0.75$ (Figs. 9d, 12a), it is found that aggregate formation is delayed in the two-way coupling approach. Hence, larger aggregates are formed only in the last section of the microchannel ($x/2R_{{\text {cyl}}}>6.0$), and only limited wall-deposited particles are observed in the channel. This difference can be explained by the fact that the two-way coupling approach is not assuming a freeze boundary condition at the wall, but allows the resuspension of deposited particles if the particle–fluid interaction is able to overcome wall adhesion. This delayed aggregate formation when advancing from the one-way to two-way coupling approach is also visualized in Fig. 13, which depicts the comparison of the percentages of aggregates of a certain size at the three intersections $x/2R_{{\text {cyl}}}=2.0, 4.0,$ and 6.0 obtained for the one- and two-way coupling approaches.

Comparing the one-way and two-way coupling approaches, an approximately 10$\%$ decrease in large aggregates ($N_{\text {c}}\ge 19$) in the first section of the microchannel is found for the CFD–DEM case. Small aggregates are observed close to the entry section for the one-way coupled case, whereas in the two-way coupled case, large aggregates are formed while flowing through the microchannel. A further increase in Reynolds numbers to ${Re}=1.5$ also exhibits the same phenomena of delayed aggregate formation. Thus, the observed trends show that high values of the Reynolds number and the adhesion parameter promote wall deposition and aggregate formation, which can finally lead to microchannel clogging.

**Fig. 14:**
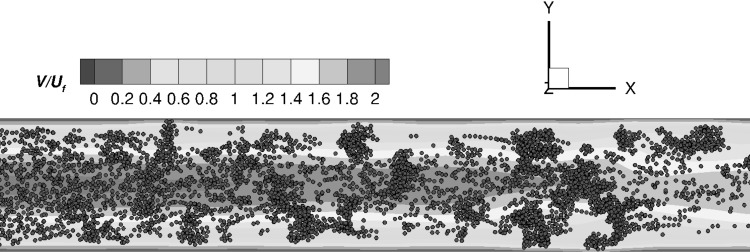
Contour plot of the instantaneous streamwise velocity (normalized with the bulk velocity $U_{\text {f}}$) in the center of the 3D CFDEM microchannel at ${Re}=1.5$ for $\phi =15{,}000$ ($t_{\text {3D}}=10$)

Furthermore, the two-way coupling approach also allows to study the effect of the particle aggregates on the fluid velocity field. Figure 14 depicts a contour of the normalized instantaneous streamwise velocity $V/U_{\text {f}}$ at the dimensionless time $t_{\text {3D}}=10$ for a Reynolds number of $Re=1.5$ ($\phi =15{,}000$).

**Fig. 15:**
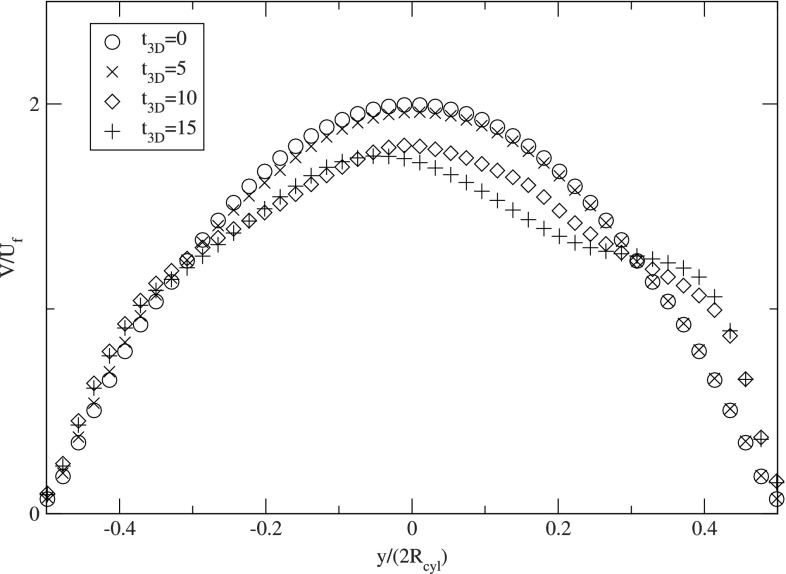
Profiles of the instantaneous streamwise fluid velocity extracted at $x/(2R_{{\text {cyl}}})=6.0$ for the 3D CFDEM microchannel at ${Re}=1.5$ and $\phi =15{,}000$

The presence of the large agglomerates significantly affects the velocity distribution in the channel, reducing the fluid velocity in the center of the microchannel resulting in increased velocities in the near wall region of the channel. This dynamic change of the streamwise velocity profile is shown in more detail in Fig. 15.

This figure depicts the profiles of the instantaneous streamwise fluid velocity extracted at $x/(2R_{{\text {cyl}}})=6.0$ at dimensionless times $t_{\text {3D}}=0$, 5, 10, and 15, respectively. At $t_{\text {3D}}=0$, the undisturbed parabolic laminar profile is observed, which is increasingly affected in the near wall region due to the aggregate generation, which results in a higher velocity close to the wall for $t_{\text {3D}}=10$ and $t_{\text {3D}}=15$. This increased near wall velocity will also lead to an increased wall shear stress, which will promote the detachment of deposited particles.

**Fig. 16:**
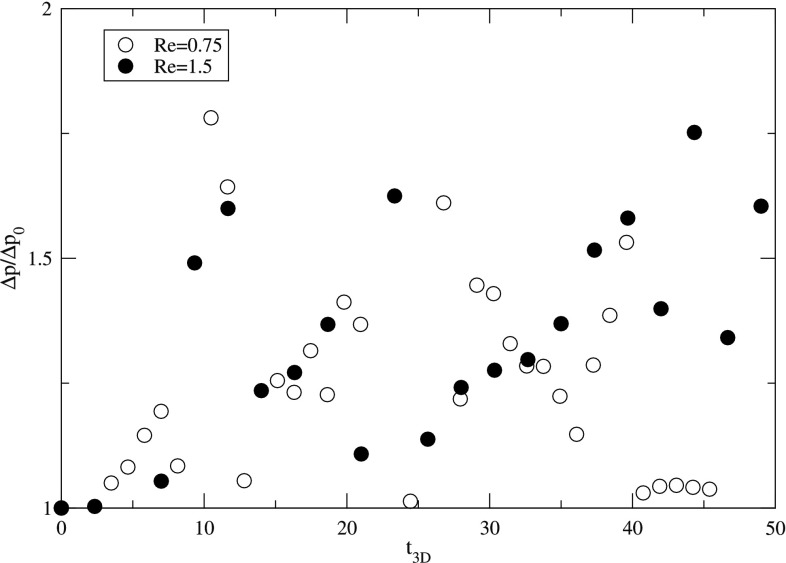
Evolution of the normalized pressure drop $\Delta p/\Delta p_0$ over time for $Re=0.75$ (open symbols) and $Re=1.5$ (closed symbols), $\phi =15{,}000$

These changes in the velocity field are also captured in the pressure drop over the computational domain. Figure 16 depicts the normalized pressure drop $\Delta p/\Delta p_0$, where $\Delta p_0$ represents the pressure drop of the developed flow without particles, for both considered Reynolds numbers.

It is observed that the pressure drop will initially ($t_{\text {3D}}<10$) increase non-linearly with time due to the presence of particles, and subsequent agglomeration and wall deposition. For longer simulation times, the pressure drop starts to fluctuate, which is explained by the formation of larger agglomerates in the bulk of the fluid flowing through the channel, leading to large disturbances of the velocity field. The finding of fluctuating pressure drops over the entire domain is in line with experimental observations of solid forming reactions (Hartman et al. 2010; Kuhn et al. 2011).

## Conclusions

In this study, we investigated the behavior of suspensions containing rigid microparticles flowing through rectangular (2D) and cylindrical (3D) microchannels using a Discrete Element Method (DEM) and a CFD–DEM coupling method. Simulations were performed at different values of the Reynolds number and the adhesion parameter, with an overall volume fraction of particles of 0.1 (2D) and 0.05 (3D). The particle–particle, particle–wall, and particle–fluid interactions were characterized in terms of statistics of aggregate formation and wall deposition.

The results reveal that particle aggregation is initiated in the near wall region of the microchannel. Furthermore, wall attachments are already observed at considerably low values of the Reynolds numbers and the adhesion parameter. As expected, increasing the value of the adhesion parameter results in larger agglomerate sizes and in their earlier formation. A further increase in the Reynolds numbers results in increased particle–aggregate, aggregate–aggregate and aggregate–wall interactions. For the condition of largest considered Reynolds numbers and adhesion parameters, large-sized aggregates are formed together with enhanced wall deposition, which finally leads to microchannel blockage. Comparing the 2D and 3D one-way coupled simulation cases, larger agglomerate sizes are observed for the 3D case at the same conditions.

Advancing to the two-way coupled CFD–DEM simulations, the influence of the particles on the fluid velocity field is characterized. Compared with the one-way coupled 3D case, a delay in wall deposition is observed, however, the overall size of the formed agglomerates is similar. The presence of large size agglomerates bridging the channel leads to the fluid bypassing them, which in turn results in increased near wall velocities and shear stress, which can resuspend attached particles from the wall. And also in the CFD–DEM simulations, we observe increased aggregation dynamics with increasing Reynolds number.

In conclusion, as the three-dimensional two-way coupling approach is able to resolve the dynamics in wall shear stress and associated delayed particle deposition, it represents the more realistic approach to capture particle–fluid dynamics in microfluidic applications and to predict clogging. As such, these CFD–DEM simulations provide the opportunity to guide experimental studies, as they allow to evaluate the clogging risk of a particular microfluidic device, and its ability to handle different particles with different adhesion parameters. Furthermore, the two-way coupling approach also enables the prediction of the optimum fluid dynamic conditions for the production of particles and agglomerates, with applications in, e.g., nanomaterial synthesis.

## Electronic supplementary material

Below is the link to the electronic supplementary material.

Supplementary material 1 (pdf 208 KB)

## Roman letters

*a*: Contact radius
$a_{0}$: Equilibrium contact radius
*C*: Number of aggregates
$$C_{\text {D}}$$: Drag coefficient
$$d_{\text {P}}$$: Particle diameter
*E*: Effective elastic modulus of colliding particles
$e_{\text {r}}$: Coefficient of restitution
$\varvec{F}_{\text {A}}$: Collision and adhesive force on the particle
$F_{\text {C}}$: Maximum particle adhesive force
$\varvec{F}_{\text {D}}$: Fluid drag force on a particle
$\varvec{F}_{\text {n}}$: Normal collision/adhesion force magnitude
$\varvec{F}_{\text {t}}$: Tangential contact force
$\varvec{F}_{\text {pf}}$: Particle–fluid interaction force
$$\varvec{F}_{\text {g}}$$: Gravitational force
$\varvec{F}^A_{\text {pf}}$: Volumetric particle–fluid interaction force
$$\varvec{f}_{d,i}$$: Drag force
$\varvec{f}_{\nabla p,i}$: Pressure gradient on each particle
$\varvec{f}_{\nabla \tau ,i}$: Viscous stress (or shear stress) on each particle
$f_{\text {add}}$: Particle adding frequency
$G^{*}$: Effective shear modulus
*H*: 2D channel width
*I*: Particle momentum of inertia
*L*: Microchannel length
$\varvec{M}_{\text {A}}$: Adhesion and collision torque on the particle
$\varvec{M}_{\text {D}}$: Fluid torque on a particle
$$\varvec{M}_{\text {t}}$$: Tangential momentum
$\varvec{M}_{\text {r}}$: Rolling friction momentum
*m*: Particle mass
$$m^*$$: Effective mass
*N*: Number of particles
$N_{\text {tot}}(t)$: Total number of particles
$\varvec{n}$: Unit normal connecting the centroids of two particles
$$\nabla p$$: Pressure gradient
$\Delta p_{0}$: Pressure drop of the developed flow without particles
$\Delta p$: Pressure drop of the developed flow with particles
*R*: Effective radius
$R_{\text {cyl}}$: Radius of the cylindrical microchannel
*r*: Particle radius
*Re*: Fluid Reynolds number
*t*: Time
$$\Delta t$$: Time step
$t_{\text {2D}}$: Dimensionless time for 2D cases
$t_{\text {3D}}$: Dimensionless time for 3D cases
$$\varvec{u}_{\text {f}}$$: Fluid velocity
$$\varvec{v}$$: Particle velocity
$\varvec{x}$: Particle position vector
$Y^{*}$: Effective Young’s modulus
$$\%\text {Aggregate}_{i}$$: Aggregate percentage

## Greek letters

$$\beta $$: Confinement ratio
$\gamma$: Adhesion surface potential
$\gamma _{\text {n}}$: Normal damping coefficient
$\gamma _{\text {t}}$: Tangential damping coefficient
$\delta _{\text {C}}$: Overlap at critical adhesive force
$\delta _{\text {N}}$: Normal overlap of particles
$\epsilon _{\text {f}}$: Fluid volume fraction in each cell
$\kappa _{\text {n}}$: Normal stiffness coefficient
$\kappa _{\text {t}}$: Tangential stiffness coefficient
$$\lambda $$: Elasticity parameter
$$\mu $$: Fluid viscosity
$\mu _{\text {r}}$: Rolling friction coefficient
$$\rho _{\text {f}}$$: Fluid density
$$\rho _{\text {p}}$$: Particle density
$$\rho _{\text {r}}$$: Density ratio
$$\sigma $$: Poisson ratio
$\tau$: Viscous force in two-way coupling approach
$$\phi $$: Adhesion parameter
$\phi _{\text {2D,3D}}$: Volume fraction of particles
$\Omega$: Particle angular velocity
