# Supplementary material for: Aggregation and clogging phenomena of rigid microparticles in microfluidics: Comparison of a discrete element method (DEM) and CFD–DEM coupling method
Source: Microfluid Nanofluidics. 2018 Aug 30;22(9):104. doi: 10.1007/s10404-018-2124-7 (PMC6190999; doi:10.1007/s10404-018-2124-7)
Supplement: Supplementary file 1 — Supplementary material 1 (pdf 208 KB) [file 10404_2018_2124_MOESM1_ESM.pdf]

Supporting Information

Aggregation And Clogging Phenomena Of Rigid Microparticles In Microfluidics

Comparison Of A Discrete Element Method (DEM) And CFD-DEM Coupling Method

Khurram Shahzad, Wouter Van Aeken, Milad Mottaghi,
Vahid Kazemi Kamyab & Simon Kuhn

KU Leuven, Department of Chemical Engineering
Celestijnenlaan 200F, 3001 Leuven, Belgium
E-mail: simon.kuhn@kuleuven.be

S1 Discrete Element Method (DEM): One-way coupling approach

In the following we briefly discuss the force terms used in Eq. (1).

S1.1 Fluid forces

The fluid force experience by each particle in laminar flow in a Newtonian fluid is given by the drag force $\boldsymbol{F}_{\mathrm{D}}$:

$$\boldsymbol{F}_{\mathrm{D}} = -3\pi d_P \mu (\boldsymbol{v} - \boldsymbol{u}_f) \tag{S1}$$

where, $\boldsymbol{u}_f$ and μ is the fluid velocity and dynamic viscosity. We consider fully developed laminar flow, and the velocity profile for 2D channel flow can be calculated as

$$u(y) = 1.5\boldsymbol{u}_f \left[1 - \left(\frac{y}{H}\right)^2\right] \tag{S2}$$

and for a 3D cylindrical channel as

$$u(r) = 2\boldsymbol{u}_f \left[1 - \left(\frac{r}{R_{\mathrm{cyl}}}\right)^2\right] \tag{S3}$$

S1.2 Collision and adhesion forces

We neglect sliding and twisting motions of interacting particles, and therefore only the normal component of adhesion and collision forces $\boldsymbol{F}_{\mathrm{A}}$ in Eq. (1) are considered,

$$\boldsymbol{F}_{\mathrm{A}} = F_{\mathrm{n}}\boldsymbol{n} \tag{S4}$$

where F_{n} represents the normal force magnitude and $\boldsymbol{n}$ is the unit vector connecting the centers of two particles:

$$\boldsymbol{n} = \frac{\boldsymbol{x}_j - \boldsymbol{x}_i}{|\boldsymbol{x}_j - \boldsymbol{x}_i|} \tag{S5}$$

where $\boldsymbol{x}_i$ and $\boldsymbol{x}_j$ are the position vectors to two generic particles i and j.

The normal force magnitude F_{n} is the sum of adhesion and elastic deformation of the particles and energy losses during the impact of the particles. The latter contribution can be neglected for very small particles [4].

During the computations we also make use of the effective radius R and the effective elastic modulus E of two generic particles i and j upon impact, which are defined as

$$\frac{1}{R} = \frac{1}{r_i} + \frac{1}{r_j} \tag{S6}$$

$$\frac{1}{E} = \frac{1 - \sigma_i^2}{E_i} + \frac{1 - \sigma_j^2}{E_j} \tag{S7}$$

where r_i and r_j are the radii of two particles with σ_i and σ_j their Poisson ratios and E_i and E_j their elastic moduli.

We consider the Johnson-Kendall-Roberts (JKR) contact model [2] to capture the aggregate generation in the flowing suspension. We assume that particle-particle collisions are limited to

their contact region, and therefore the contact upon particle collision is computed only by the instantaneous forces occurring at that time but not by their previous history. The normal elastic force magnitude F_n and particle normal overlap δ_{N} can be calculated as a function of the contact region radius a [1, 2]:

$$\frac{F_n}{F_{\mathrm{C}}} = 4\left(\frac{a}{a_0}\right)^3 - 4\left(\frac{a}{a_0}\right)^{3/2} \tag{S8}$$

where F_{C} is the critical force which can be calculated using the Van der Waals surface potential energy of the particle γ

$$F_{\mathrm{C}} = 3\pi\gamma R \tag{S9}$$

The contact region radius a is linked to the particle normal overlap δ_{N} via

$$\delta_{\mathrm{N}} = r_i + r_j - |\boldsymbol{x}_i - \boldsymbol{x}_j| \tag{S10}$$

and by the following relation between δ_{N} and critical overlap δ_{C}

$$\frac{\delta_{\mathrm{N}}}{\delta_{\mathrm{C}}} = 6^{1/3}\left[2\left(\frac{a}{a_0}\right)^2 - \frac{4}{3}\left(\frac{a}{a_0}\right)^{1/2}\right] \tag{S11}$$

in which the term δ_{C} provides the critical overlap distance between two particles

$$\delta_{\mathrm{C}} = \frac{a_0^2}{2(6)^{1/3}R} \tag{S12}$$

The equilibrium contact radius a_0 used in above Eqs. (S8), (S11) and (S12) is the contact region suggested in the JKR model [2] and is expressed as:

$$a_0 = \left(\frac{9\pi\gamma R^2}{E}\right)^{1/3} \tag{S13}$$

To speed-up the simulations pre-computation was performed for Eqs. (S8) and (S11). The results are stored in a look-up table, i.e., both $\delta_{\mathrm{N}}/\delta_{\mathrm{C}}$ and $F_{\mathrm{N}}/F_{\mathrm{C}}$ are solved for variuos a/a_0 values. During the time-stepping computation the generated $\delta_{\mathrm{N}}/\delta_{\mathrm{C}}$ value is then used (with appropriate interpolation) to extract the corresponding value of $F_{\mathrm{N}}/F_{\mathrm{C}}$ from the table. We also adopt a neighbor-list algorithm approach, in such a way that particles can experience only collision/adhesion forces at a certain distance from each other. For this, the entire channel is divided into small blocks of size $d + \delta_{\mathrm{C}}$, and the collision/adhesion force computation is only performed if the particles are present in three consecutive blocks.

S2 CFD-DEM: Two-way coupling approach

Table S1: Equations to calculate the forces and parameters in the DEM part of the CFD-DEM coupling. The indices i and j denote two particles.

**Force / parameter**	**Equation**
Normal contact force	$\boldsymbol{F}_{n,ij} = [\kappa_n \boldsymbol{\delta}_n - \gamma_n (\boldsymbol{v}_{ij} \cdot \boldsymbol{n}_{ij})]\boldsymbol{n}_{ij}$
Tangential contact force	$\boldsymbol{F}_{t,ij} = -min[\mu \lvert \boldsymbol{F}_{n,ij} \rvert, \kappa_t (\boldsymbol{\delta}_t \cdot \boldsymbol{t}_{ij}) - \gamma_t (\boldsymbol{v}_t \cdot \boldsymbol{t}_{ij})]\boldsymbol{t}_{ij}$
Normal stiffness coefficient	$\kappa_n = \frac{4}{3} Y^* \sqrt{R^* \boldsymbol{\delta}_n}$
Tangential stiffness coefficient	$\kappa_t = 8G^* \sqrt{R^* \boldsymbol{\delta}_n}$
Normal damping coefficient	$\gamma_n = -2\sqrt{\frac{5}{6}} \frac{ln(e_r)}{\sqrt{ln^2(e_r)+\pi^2}} \sqrt{\frac{2}{3}\kappa_n m^*}$
Tangential damping coefficient	$\gamma_t = -2\sqrt{\frac{5}{6}} \frac{ln(e_r)}{\sqrt{ln^2(e_r)+\pi^2}} \sqrt{\kappa_t m^*}$
Tangential forces torque	$\boldsymbol{M}_{\mathrm{t}} = \boldsymbol{r}_i \times (\boldsymbol{F}_t)$
Rolling friction torque	$\boldsymbol{M}_{\mathrm{r}} = -\mu_r \lvert \boldsymbol{F}_n \rvert \frac{\omega}{\lvert\omega\rvert} R$
Effective radius	$\frac{1}{R} = \frac{1}{r_i} + \frac{1}{r_j}$
Effective mass	$\frac{1}{m^*} = \frac{1}{m_i} + \frac{1}{m_j}$
Effective shear modulus	$\frac{1}{G^*} = \frac{2(2+\sigma_i)(1-\sigma_i)}{Y_i} + \frac{2(2+\sigma_j)(1-\sigma_j)}{Y_j}$
Effective Young's modulus	$\frac{1}{Y^*} = \frac{\left(1-\sigma_i^2\right)}{Y_i} + \frac{\left(1+\sigma_j^2\right)}{Y_j}$
Pressure gradient term	$-\frac{\pi}{6} d_p^3 \nabla p$
Viscous force term	$-\frac{\pi}{6} d_p^3 \nabla \cdot \boldsymbol{\tau}$
Di Felice drag force	$\boldsymbol{F}_{\mathrm{D}} = C_{\mathrm{D}} \frac{\rho(\boldsymbol{v}-\boldsymbol{u}_f)^2}{2} \frac{\pi d_p^2}{4} f(\epsilon_f)$<br>$C_{\mathrm{D}} = \left(0.63 + \frac{4.8}{Re_p^{0.5}}\right)^2 f(\epsilon_f)$<br>$f(\epsilon_f) = \epsilon_f^{-(\alpha+1)}$,<br>$\alpha = 3.7 - 0.65exp[-(1.5 - log\mathrm{Re}_p)^2/2]$,<br>$\mathrm{Re}_p = \frac{\rho_f d_{p,i} \lvert \mathbf{u} - \mathbf{v}_i \rvert}{\mu}$

S3 CFD-DEM coupling strategy

To apply the CFD-DEM coupling, both the DEM solver (LIGGGHTS) and an OpenFOAM CFD solver are run in parallel. The data exchange between LIGGGHTS and OpenFOAM is done after a predefined number of time-steps (here: 10 DEM time-steps). The coupling approach is based on several computation steps [3]:

1. The particle positions and velocities are calculated by the DEM solver (LIGGGHTS)
2. The particle positions and velocities are transferred to the CFD solver (OpenFOAM)
3. The corresponding cell according to the provided position of each particle in the CFD mesh is found
4. The particle volume fraction and mean particle velocity is calculated for each cell
5. The fluid forces acting on each particle are calculated according to the particle volume fraction in that cell
6. The particle-fluid momentum exchange terms are computed by averaging all the particle based forces in each CFD cell
7. The fluid velocity is computed by using the local volume fraction and momentum exchange terms
8. The data are returned to the DEM solver for the next time step after calculating the fluid forces on each particle
9. The entire routine is repeated starting from Eq. (3)

References

[1] A. Chokshi, A. G. G. M. Tielens, and D. Hollenbach. Dust coagulation. *Astrophys. J.*, 407:806–819, 1993.

[2] K. L. Johnson, K. Kendall, and A. D. Roberts. Surface energy and the contact of elastic solids. *Proc. R. Soc. A Math. Phys. Eng. Sci.*, 324:301–313, 1971.

[3] C. Kloss, C. Goniva, A. Hager, S. Amberger, and S. Pirker. Models, algorithms and validation for opensource DEM and CFD–DEM. *Progress in Computational Fluid Dynamics, An International Journal*, 12(2-3):140–152, 2012.

[4] K. Shahzad, G. DAvino, F. Greco, S. Guido, and P. L. Maffettone. Numerical investigation of hard-gel microparticle suspension dynamics in microfluidic channels: Aggregation/fragmentation phenomena, and incipient clogging. *Chemical Engineering Journal*, 303:202–216, 2016.
